# Role of the YWHAG gene mutations in Developmental and Epileptic Encephalopathy

**DOI:** 10.3389/fnins.2025.1641250

**Published:** 2025-08-15

**Authors:** Violet Vilmont, Richard S. Nowakowski, Yi Zhou

**Affiliations:** Department of Biomedical Sciences, Florida State University College of Medicine, Tallahassee, FL, United States

**Keywords:** YWHAG mutation, 14-3-3γ protein, developmental and epileptic encephalopathy, epilepsy, seizure, neuronal hyperexcitability, DEE, 14-3-3 protein family

## Abstract

Developmental and Epileptic Encephalopathy (DEE) is a severe neurological condition characterized by epileptic seizures and cognitive developmental impairments. Mutations in the YWHAG gene, which encodes the 14-3-3γ protein, are implicated in DEE. Predominantly expressed in the brain, 14-3-3γ regulates various cellular processes, forming homodimers or heterodimers with other isoforms. It binds to phosphorylated sites on target proteins, influencing their activity, stability, or cellular localization. This review evaluates the association between YWHAG mutations and DEE, the mechanisms by which 14-3-3γ influences neuronal function, and potential therapeutic interventions. YWHAG mutations, often *de novo*, lead to a variety of epilepsy phenotypes, from febrile seizures to severe epileptic encephalopathies. Loss-of-function mutations disrupt neuronal homeostasis, contributing to epilepsies and cognitive dysfunction. Specific missense mutations in the 14-3-3γ, such as Arg132Cys, significantly impair the protein’s binding affinity and are associated with a severe DEE. These mutations impact the function and stability of 14-3-3γ, affecting its interaction with ion channels and proteins, thereby contributing to neuronal hyperexcitability and impaired development. Understanding the involvement of YWHAG in DEE can provide insights into targeted treatments that address both the epileptic and developmental components of the disorder.

## 1 Introduction

Developmental and Epileptic Encephalopathies (DEEs) are a group of severe neurological disorders characterized by early-onset epilepsy, developmental delays, and often progressive cognitive and behavioral impairments. These disorders are highly heterogeneous, both clinically and genetically, presenting significant challenges for diagnosis and treatment (Guerrini et al., 2023). A key feature of DEEs is the disruption of normal brain development and function, often driven by mutations in genes critical for synaptic transmission, neuronal signaling, and network homeostasis (Guella et al., 2017). The 14-3-3 protein family plays a significant role in maintaining neuronal health, regulating synaptic city, and supporting cortical development (Foote and Zhou, 2012). Within this family, the dysfunction of 14-3-3γ isoform has emerged as a key factor in the pathophysiology of DEEs (Kanani et al., 2020). This review will explore the connection between 14-3-3γ and DEE, as well as the potential mechanisms.

The 14-3-3 proteins are a highly conserved family of regulatory molecules involved in a wide range of cellular processes, including cell cycle control, signal transduction, and apoptosis (Aitken et al., 1995; Shen et al., 2003). In the brain, these proteins are particularly abundant, supporting critical functions such as neuronal migration, axonal growth, synaptic development, and plasticity (Ferl et al., 2002; Foote and Zhou, 2012). Among the seven isoforms of the 14-3-3 family, 14-3-3γ, encoded by the YWHAG gene, has been specifically implicated in neurodevelopment (Cho and Park, 2020; Huang et al., 2022). Mutations or dysregulation of YWHAG have been associated with intellectual disabilities, autism spectrum disorders, and a spectrum of epilepsy phenotypes, including DEEs (Guella et al., 2017; Ye et al., 2021; Yi et al., 2022). Despite growing evidence of its importance, the molecular mechanisms by which 14-3-3γ dysfunction contributes to DEEs remain poorly understood and further investigation is needed.

Recent studies using 14-3-3γ-deficient animal models have provided insights into its role in neurodevelopment and disease. For instance, these models have demonstrated the importance of 14-3-3γ in cortical development, synaptic plasticity, and maintaining excitatory-inhibitory balance in neural networks (Foote et al., 2015; Roy et al., 2021). Loss of 14-3-3 function has been shown to disrupt NMDA receptor localization and function (Qiao et al., 2014; Lee et al., 2021), impair neuronal migration (Cornell and Toyo-oka, 2017), and lead to behavioral phenotypes consistent with neuropsychiatric and epileptic disorders (Kim et al., 2019; Logue et al., 2024). Such findings demonstrate the potential of targeting 14-3-3γ-related pathways for therapeutic development in DEEs and related conditions.

## 2 The 14-3-3γ isoform

### 2.1 Overview of 14-3-3 protein family

The 14-3-3 proteins are a highly conserved family of regulatory molecules expressed in all eukaryotes, playing critical roles in various cellular processes. The historical naming of the 14-3-3 proteins originates from their elution and migration patterns observed during DEAE-cellulose chromatography and starch gel electrophoresis. These proteins were identified in the 14th fraction of bovine brain homogenate on DEAE-cellulose and migrated to position 3.3 in the starch electrophoresis gel, giving rise to their name (Moore and McGregor, 1965; Moore, 1969).

In humans, the 14-3-3 protein family consists of seven isoforms, each encoded by a distinct gene: YWHAB/YWHAA (14-3-3*β*/14-3-3*α*), YWHAG (14-3-3γ), YWHAE (14-3-3ε), YWHAH (14-3-3η), SFN or YWHAS (14-3-3σ), YWHAQ (14-3-3τ in humans, 14-3-3θ in mice), and YWHAZ/YWHAD (14-3-3*ζ*/14-3-3*δ*). Isoforms α and δ are the phosphorylated forms of β and ζ, respectively (Aitken et al., 1995). These seven isoforms are evolutionarily conserved across mammalian species, including mice, which possess the same set of seven genes and the encoded proteins (Table 1).

**Table 1:** The seven isoforms of 14-3-3 protein family—in humans and in mice.

Gene name (*human*)	YWHAB or YWHAA	YWHAG	YWHAE	YWHAH	YWHAS or SFN	YWHAQ	YWHAZ or YWHAD
Gene name (*mouse*)	Ywhab or Ywhaa	Ywhag	Ywhae	Ywhah	Ywhas or Sfn	Ywhaq*	Ywhaz or Ywhad
Protein isoform	14-3-3β (beta) or 14-3-3α (alpha) if phosphorylated	14-3-3γ (gamma)	14-3-3ε (epsilon)	14-3-3η (eta)	14-3-3σ (sigma)	14-3-3τ (tau) in humans or 14-3-3θ (theta) in mice*	14-3-3ζ (zeta) or 14-3-3δ (delta) if phosphorylated
Salt bridges	3	2	1	2	3	3	3
Base pairs/amino acids (*human*)	3020/246	3705/247	2052/255	1751/246	1308/248	2196/245	5011/245
Base pairs/amino acids (*mouse*)	3013/246	3520/247	2100/255	1764/246	1613/248	2197/245	3288/245

The 14-3-3 proteins exist as either homo- or hetero-dimers (Chaudhri et al., 2003). There are 28 possible 14-3-3 dimer combinations: 7 homodimers and 21 heterodimers. The relative abundance and types of dimers vary across cellular locations, tissues, and organs, and this diversity in dimerization influences their functional roles. Dimerization is critical for 14-3-3 function, as it stabilizes their structure and creates the proper conformation necessary for binding target proteins (Shen et al., 2003). The functionality of these dimers depends on the specific isoforms involved, their interaction partners, and the cellular context.

### 2.2 Structural characteristics and isoform-specific features of the 14-3-3 family

The seven isoforms of the 14-3-3 protein family share a high degree of structural similarity, yet each exhibits unique features that influence its specific functions within the cell. These differences include variations in expression patterns, binding partners, and structural stability (Gardino et al., 2006; Obsilova and Obsil, 2022). Sequence analyses reveal a homology range of 69–88% among the isoforms (Sengupta et al., 2020), with conserved regions forming the hallmark cup-shaped grove crucial for target protein interactions (Huang et al., 2022). This characteristic groove binds phosphoserine- or phosphothreonine-containing motifs on target proteins, facilitating their regulatory functions (Obsil and Obsilova, 2011). While the overall dimerization region is conserved across isoforms, subtle differences in amino acid sequences at inter-subunit contact regions can impact dimer stability. For example, the number of stabilizing salt bridges varies among homodimers, with one in 14-3-3ε, two in 14-3-3γ and *η*, and three in 14-3-3β, *ζ*, *σ*, and *τ* (Gardino et al., 2006).

Each 14-3-3 monomer has nine *α*-helices arranged in an antiparallel fashion, forming an L-shaped structure (Ferl et al., 2002). Comparisons of crystal structures among human isoforms reveal consistent overall architecture, with minor differences in subunit angles, loop lengths, and α-helix lengths—most notably helices H3, H4, and their connecting loop (Yang et al., 2006). Flexible loop regions, such as those connecting helices H3-H4 and H8-H9, often appear disordered in crystal structures, suggesting high adaptability in protein interactions (Obsilova and Obsil, 2022).

Key structural elements include the helices H3, H5, H7, and H9, which contribute to the formation of an amphipathic groove responsible for target peptide binding. Charged and polar amino acids dominate helices H3 and H5, while H7 and H9 are enriched with hydrophobic residues. This concave groove allows each monomer to bind one phosphopeptide, enabling a dimer to simultaneously interact with two phosphorylated sites (Ferl et al., 2002; Gogl et al., 2021).

The binding interactions follow two primary conserved motif sequences: RSX(pS/pT)XP (mode I) and RXPhiX(pS/pT)XP (mode II), which define the distinct patterns of 14-3-3 protein interactions with their targets (Coblitz et al., 2005).

### 2.3 The function of 14-3-3γ

The 14-3-3γ protein, encoded by YWHAG gene, is a crucial regulatory molecule involved in diverse cellular processes. Located on chromosome 7q11.23 in humans, YWHAG produces a protein 247 amino acids in length, identical in size to its mouse ortholog encoded by the Ywhag gene on chromosome 5. Comparative analyses reveal an 88.04% nucleotide sequence identity between the human and mouse genes, maintaining evolutionary conservation of 14-3-3γ across species. The 14-3-3γ forms either homo- or hetero-dimers with other 14-3-3 isoforms, exhibiting preferential pairing with 14-3-3ε (Chaudhri et al., 2003; Huang et al., 2022).

A defining characteristic of 14-3-3γ is its ability to bind phosphorylated target molecules via a conserved ligand-binding groove, which is formed when two 14-3-3 subunits dimerize. This interaction influences various cellular functions, including regulating protein activity, stability, and localization. Through these interactions, 14-3-3γ plays a central role in signal transduction, modulating pathways critical for cellular responses to environmental cues (Cho and Park, 2020). Additionally, it is implicated in cell cycle regulation, where it influences processes such as cell division and growth, and in apoptosis, where its interactions determine cellular survival or programmed cell death (Qian Chen and Cheung Hoi Yu, 2002).

14-3-3γ also contributes to cellular stress responses, enabling cells to adapt to environmental changes by modulating stress-responsive proteins. Notably, it interacts with key signaling molecules like RAF1 and protein kinase C, highlighting its involvement in complex signal transduction networks (Pagliuso et al., 2016; Xu et al., 2021). These interactions are supported by evidence showing that 14-3-3γ exhibits the highest equilibrium binding affinity among the seven 14-3-3 isoforms, engaging with over 400 out of 547 identified phosphopeptide-binding proteins (Gogl et al., 2021).

Functional diversity of 14-3-3γ is further demonstrated by a yeast two-hybrid study that identified 170 unique protein interactions. These proteins span various biological functions: 45% are involved in cellular communication and signal transduction, 15% in nucleic acid synthesis and processing, 10% in cellular organization, and smaller percentages in energy metabolism and other processes (Jin et al., 2004).

**Figure 1:**
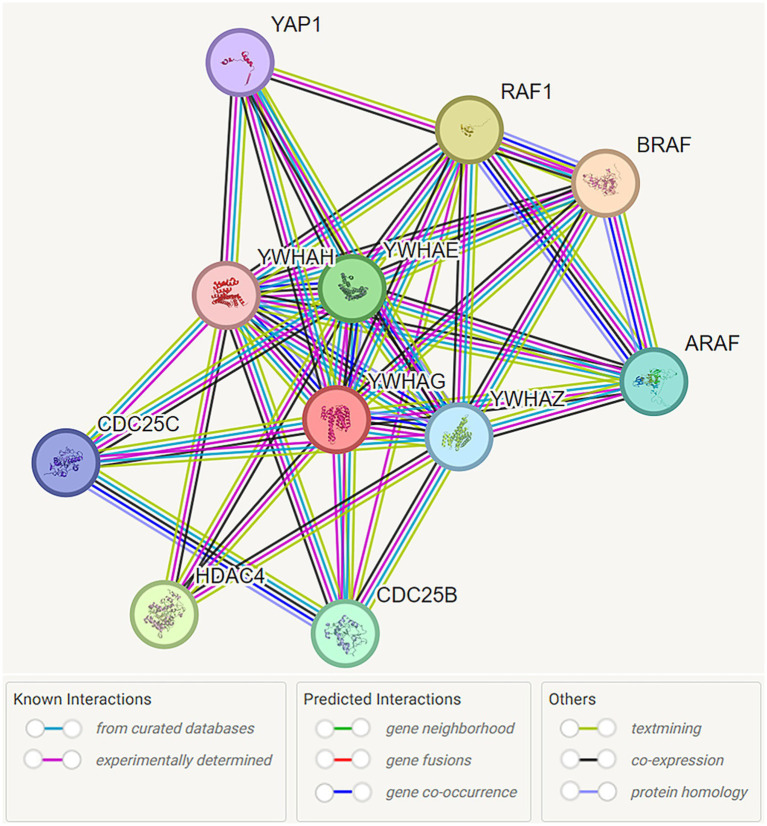
Functional interaction network of 14-3-3γ encoded by YWHAG. Network nodes are labeled with the name of the individual genes which encode the represented proteins. Protein interactions are represented by color coded lines, based on known and predicted interactions, as indicated by the legend. Source: https://stringdb.org/cgi/network?taskId=bMYGzw1kOtuv&sessionId=bMliKFIeKj5g. Screenshot image obtained from the STRING database (string-db.org). Licensed under the Creative Commons Attribution 4.0 International License (CC BY 4.0).

According to the STRING database curated by the Global Biodata Coalition and ELIXIR, the network illustrates predicted functional associations between the YWHAG gene and other genes based on co-expression, shared pathways, and experimental evidence, rather than direct physical, protein–protein interactions (Figure 1). These gene-level interactions, including those with other 14-3-3 isoforms (YWHAE, YWHAZ, YWHAH) and various signaling pathway components, reflect the broad role of YWHAG in maintaining cellular homeostasis.

### 2.4 The expression and localization of 14-3-3γ

The 14-3-3 protein family constitutes approximately 1% of the total soluble proteins in the brain (Foote and Zhou, 2012), reflecting its significant role in neural function. Among them, 14-3-3γ is particularly abundant in the brain but is also expressed across various tissues, highlighting its versatile roles in neuronal development, synaptic activity, and cellular signaling. Although the precise contribution of 14-3-3γ to the total pool of 14-3-3 proteins is not well-defined, its expression varies significantly among tissue types and cellular environments.

**Figure 2:**
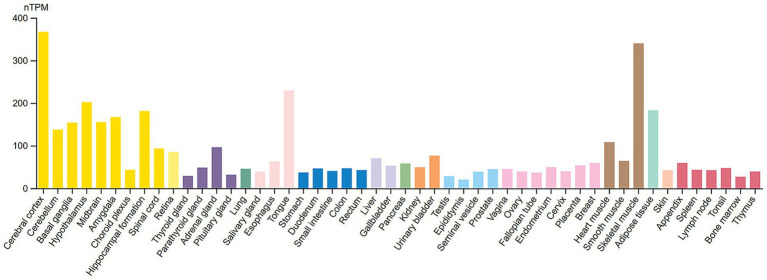
RNA tissue specificity expression of 14-3-3γ. Normalized RNA expression levels (nTPM) shown for 55 tissue types. Color coding is based on tissue groups, each consisting of tissues with functional features in common. RNA tissue specificity expression is enhanced in brain (yellow bars) and skeletal muscle cells (brown bars). Source: https://www.proteinatlas.org/ENSG00000170027-YWHAG/tissue. Screenshot image obtained from the Human Protein Atlas (proteinatlas.org). Licensed under the Creative Commons Attribution-ShareAlike 4.0 International License (CC BY-SA 4.0).

**Figure 3:**
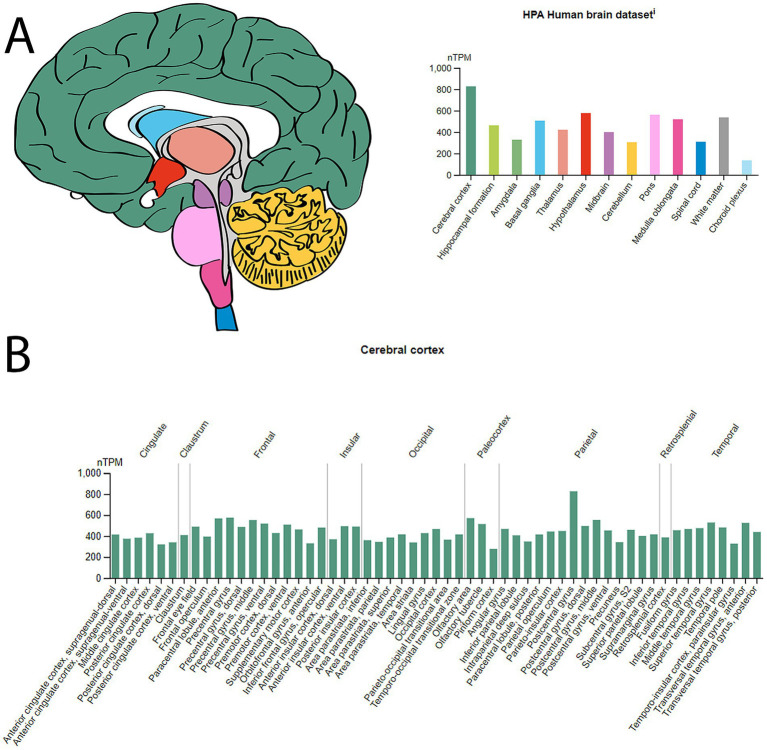
Brain RNA expression of 14-3-3γ. **(A)** Normalized RNA expression levels (nTPM) shown for the 13 brain regions. Color coding is based on brain region. The bar shows the highest expression among the brain subregions included. HPA, Human Protein Atlas. **(B)** Normalized RNA expression levels (nTPM) shown for the Cerebral Cortex. The highest expression of 14-3-3γ within the Cerebral Cortex is in the Postcentral Gyrus. Source: https://www.proteinatlas.org/ENSG00000170027-YWHAG/brain. Screenshot image obtained from the Human Protein Atlas (proteinatlas.org). Licensed under the Creative Commons Attribution-ShareAlike 4.0 International License (CC BY-SA 4.0).

The Human Protein Atlas (HPA) data shows the expression patterns of the 14-3-3γ transcripts across various tissues and brain regions. Its RNA expression levels are reported for 55 tissue types, with notably enhanced expression in the brain and skeletal muscle cells (Figure 2). In addition, normalized 14-3-3γ RNA expression levels (nTPM) are provided for 13 brain regions, showing the highest levels within the cerebral cortex region, with the postcentral gyrus subregion exhibiting the highest expression of 14-3-3γ (Figure 3).

In the brain, 14-3-3γ shows predominant expression in neurons at mRNA levels compared to other isoforms, which display more uniform distribution across different cell types (Watanabe et al., 1993). In regions such as the striatum and substantia nigra, neuronal localization of 14-3-3γ is concentrated in the soma, dendrites, and axons (Cho et al., 2023). Astrocytes in ischemic conditions also show heightened levels of 14-3-3γ, where its upregulation enhances astrocyte survival during ischemia and its depletion leads to increased astrocyte apoptosis (Chen et al., 2003, 2005). In oligodendrocytes, a deficiency in 14-3-3γ has been linked to demyelination and increased vulnerability to inflammatory insults (Cho and Park, 2020).

Beyond its brain-specific roles, 14-3-3γ exhibits diverse subcellular localization and interactions. While most 14-3-3 isoforms are found in the cytoplasm, intracellular organelles, and plasma membrane, 14-3-3γ is primarily localized to the nucleus, where it forms distinct particles but avoids the nucleoli (Abdrabou et al., 2020). It has also been detected in centrosomes, with its loss resulting in centrosome amplification, a phenomenon implicated in cellular instability (Mukhopadhyay et al., 2016). The unique distribution of 14-3-3γ within the cell contributes to its regulation of various target proteins, influencing their activity, stability, and localization.

A recent study has shown that pathogenic YWHAG mutations cause nuclear relocalization of 14-3-3γ and impair its ability to bind phosphorylated targets, disrupting normal cytoplasmic signaling functions (Larasati et al., 2025). To address this, a high-throughput drug screening approach tested ~3,000 approved compounds for their ability to restore 14-3-3γ–phosphotarget interactions. While no definitive therapeutic was validated, *in vitro* assays identified nafamostat as a potential candidate, highlighting the feasibility of small-molecule strategies to rescue 14-3-3γ function in YWHAG-related disorders (Larasati et al., 2025).

## 3 Association of YWHAG mutation with DEE

### 3.1 DEE overview

Developmental and Epileptic Encephalopathy (DEE) represents a group of severe neurological disorders marked by early-onset epilepsy, developmental delays, and cognitive impairments (Guella et al., 2017). The clinical features include severe epileptic seizures that begin early in life and developmental impairments that often worsen over time (Guerrini et al., 2023).

Epileptic activity in DEE may not directly cause developmental delays but frequently accelerates cognitive regression. Both developmental delay (DE) and epileptic encephalopathy (EE) stem from the same underlying developmental issues, compounding severity of the disorder. The DE component reflects delays arising from the fundamental developmental problem rather than seizure activity. In contrast, the EE component, characterized by frequent seizures and abnormal EEG patterns, exacerbates developmental challenges.

**Figure 4:**
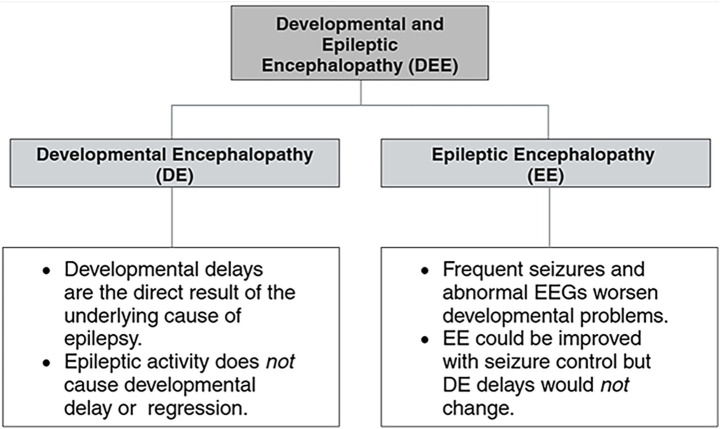
Flowchart distinction between Developmental and Epileptic Components of DEE. Created in Biorender.

While controlling seizures may mitigate the impact of EE, it does not address the developmental delays caused by the initial insult (Figure 4). DEE is therefore more severe than many other forms of epilepsy, as it impacts brain function broadly, leading to developmental delays, intellectual disabilities, and behavioral challenges.

### 3.2 Genetic causes of DEE

DEE can arise from several genetic mutations that disrupt normal brain function. According to the National Institute of Health’s Genetic Testing Registry, DEE has been linked to 118 different genes, with this number continuing to grow as research advances. These mutations often target genes involved in ion channel function, neurotransmitter receptors, and synaptic proteins.

Many cases of DEE are associated with mutations in genes affecting ion channel functions. Various DEE disorders are named in the order their associated genes are discovered, starting from DEE1 and continuing to latest DEE115. For example, sodium channel mutations such as SCN1A (DEE6A), SCN2A (DEE6B), and SCN8A (DEE13) have been linked to distinct DEE subtypes. Similarly, potassium channel mutations in KCNQ2 (DEE7) and KCNT1 (DEE14), as well as calcium channel mutations like CACNA1A (DEE42), have been implicated in DEE pathophysiology. Mutations in neurotransmitter receptor genes, such as GABRA1 (DEE19), GABRB3 (DEE43), and GRIN2D (DEE46), also contribute to DEE, further highlighting the disorder’s molecular diversity. Additionally, mutations in genes associated with synaptic proteins and transcription factors, including STXBP1 (DEE4), CDKL5 (DEE2), and CHD2 (DEE94), expand the genetic landscape of DEE.

Among these, the YWHAG gene was the 56th gene identified to be associated with DEE, leading to the DEE56 classification. Mutations in YWHAG, which encodes the 14-3-3γ protein, result in a spectrum of symptoms that can include seizures, developmental delays, and behavioral challenges. The specific features and severity of DEE56 vary among affected individuals, emphasizing the complexity of its clinical presentation (Epi4K Consortium and Epilepsy Phenome/Genome Project, 2013).

### 3.3 YWHAG mutation types and clinical phenotypic variability

**Figure 5:**
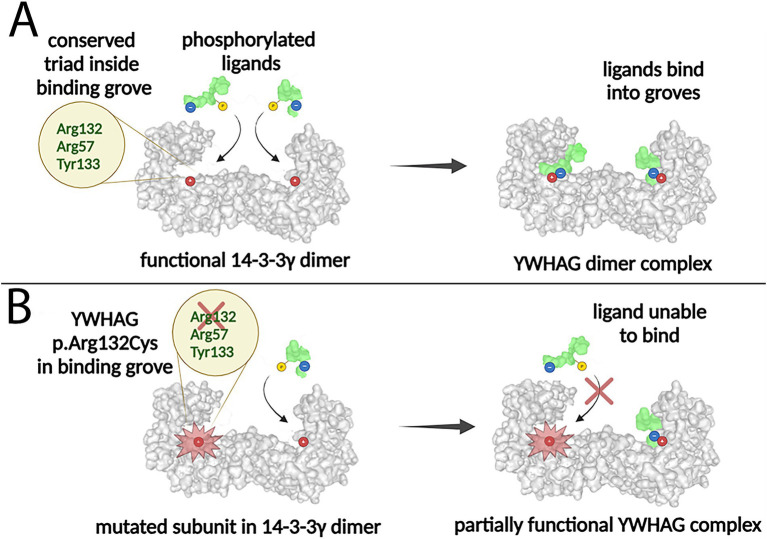
Binding of functional 14-3-3γ dimer to phosphorylated ligands and disruption by the YWHAG missense mutation. **(A)** Each functional subunit of the 14-3-3γ dimer contains an Arg132-Arg57-Tyr133 sequence that creates a stabilized, positively charged groove. This groove binds to negatively charged phosphorylated ligands, facilitating the formation of a YWHAG complex. **(B)** The missense Arg132Cys mutation in the YWHAG gene replaces the 132nd amino acid, Arginine, with Cysteine. This alteration disrupts bond formation between the 14-3-3γ dimer and phosphorylated ligands, as the mutated 14-3-3γ loses the stabilizing Arg132-Arg57-Tyr133 groove. Created in Biorender.

Mutations in the YWHAG gene, which encodes the 14-3-3γ protein, disrupt its regulatory functions by impairing interaction with phosphorylated ligands. Most identified YWHAG mutations occur within the 14-3-3γ binding groove and are missense mutations, which introduce single amino acid substitutions that alter the structure and function of 14-3-3γ (Figure 5). The pathogenicity of these missense variants stems from their disruption of the positively charged binding groove, which normally stabilizes interactions with phosphorylated ligands.

Many YWHAG mutations associated with DEEs occur at primary interaction sites within the Arg132-Arg57-Tyr133 triad of the binding groove (Ye et al., 2021). This structural motif is responsible for stabilizing the negatively charged phosphopeptides of target proteins through hydrogen bonding and electrostatic interactions (Yang et al., 2006). Missense mutations affecting these key residues, such as Arg57Gly, Arg57Cys, Tyr133Ser, and Arg132Cys, alter the groove’s electrostatic properties and compromise its ability to interact with phosphorylated targets (Skjevik et al., 2014). Several other missense mutations, including Glu15Ala, Asp129Glu, Leu177Ile, and Asn178Asp., have been associated with developmental and epileptic encephalopathy (DEE). Additionally, mutations such as Glu15Ala impair dimerization, a key structural requirement for 14-3-3γ regulatory function (Valente et al., 2012). Other missense mutations, such as Lys50Gln, are linked to Autism Spectrum Disorder (ASD), while Lys125Glu has been implicated in Febrile Seizures (FS) and Myoclonic Seizures (MS) (Ye et al., 2021). More recently, Arg57His was identified as another YWHAG variant contributing to DEE (Gheorghita et al., 2023).

**Figure 6:**
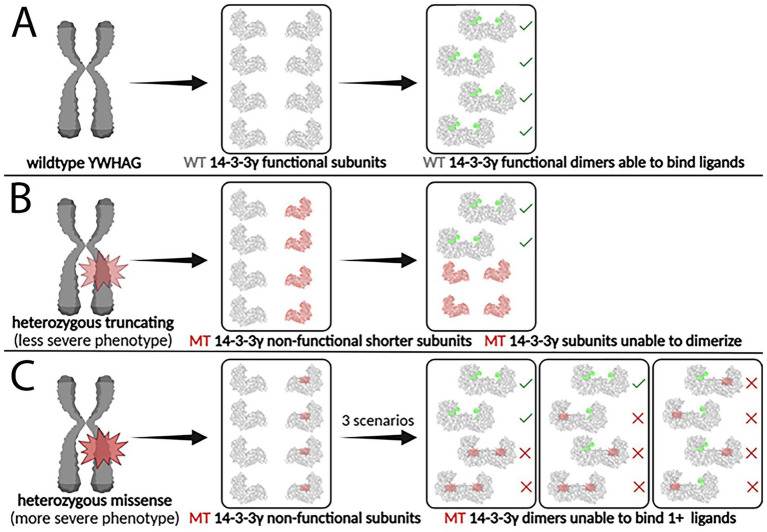
Impact of heterozygous truncating and missense YWHAG mutations on 14-3-3γ dimer formation and function. **(A)** The wildtype YWHAG gene produces normal 14-3-3γ subunits, which assemble into functional 14-3-3γ dimers, able to bind phosphorylated ligands. **(B)** A truncating mutation produces non-functional mutant 14-3-3γ subunits that are shorter and smaller, which are unable to dimerize and to bind phosphorylated ligands. **(C)** A missense mutation produces non-functional mutant 14-3-3γ subunits that are the same size as the wildtype subunits, which are able to dimerize but unable to bind two phosphorylated ligands. WT, wildtype; MT, mutant. Created in Biorender.

In contrast, truncating mutations, such as Arg42Ter, introduce a premature stop codon, leading to production of a shortened, nonfunctional protein incapable of dimerizing (Figure 6). These mutations, though less common, result in functional haploinsufficiency, as only one functional gene copy remains available for 14-3-3γ dimer formation. Individuals with truncating mutations typically exhibit milder phenotypes compared to those with missense mutations. Conversely, dominant-negative mutations, often seen in missense variants, produce mutant monomers that incorporate into dimers but impair their ability to regulate phosphorylated targets (Figure 6).

A comparison of two mutations, Arg42Ter (truncating) and Lys125Glu (missense), highlights their distinct clinical outcomes. Individuals with Arg42Ter present with relatively mild symptoms due to reduced 14-3-3γ protein levels, while those with Lys125Glu, which disrupts 14-3-3γ’s ability to bind target ligands, experience more severe neurological dysfunction (Ye et al., 2021). However, both mutations have shown responsiveness to Valproate (VPA or valproic acid), an anticonvulsant or an antiepileptic drug (AED) used to manage various seizure disorders. This suggests that pharmacological treatments targeting downstream effects of YWHAG dysfunction may provide therapeutic benefit (Kanani et al., 2020).

A recent study analyzed 24 individuals with pathogenic or likely pathogenic YWHAG variants, including 21 new cases (Cetica et al., 2024). Most patients had early-onset epilepsy within the first 2 years of life, with phenotypes ranging from generalized epilepsy to DEE. Intellectual disability (96%), behavioral disorders (75%), and other neurological features (54%) were common. Seizure control was achieved in just over half of the cohort. Missense variants in the ligand-binding domain were more frequently associated with DEE than truncating or other missense variants, suggesting a possible dominant-negative effect. Arg57 and Arg132 were identified as recurrent mutational hotspots (Cetica et al., 2024).

Another recent study reported 12 new DEE56 cases and reviewed 27 previously published cases with YWHAG mutations (Amato et al., 2024). Early-onset febrile and afebrile seizures were common, along with varying degrees of psychomotor delay. Most individuals had mild intellectual disability, and some showed comorbid autism, ADHD, or movement disorders such as ataxia and tremor. VPA showed partial efficacy in several patients. The study also identified a novel in-frame deletion Asn212_Ser215del, expanding the genotypic spectrum (Amato et al., 2024). Another novel variant, Arg125His, was also described, further broadening the known mutation types in YWHAG-related disorders (Amato et al., 2024). Lastly, an additional study further characterized the phenotypic spectrum of YWHAG-related epilepsy by analyzing 15 individuals from a Chinese cohort alongside 40 previously published cases (Tan et al., 2025). The authors identified several novel YWHAG variants, including three frameshift mutations (Thr31Aspfs*5, Ile63Thrfs*3, Ser102Alafs*7), seven missense mutations (Arg132His, Arg132Gly, Tyr133Asn, Tyr133Cys, Ser180Tyr, Ala193Val, Glu207Lys), and one truncating nonsense variant (Trp233Ter). Seven of these variants were confirmed to be *de novo*. Overall, 86.7% of cases in the cohort carried *de novo* mutations, most of which were missense variants. Clinical presentations ranged from isolated febrile seizures to severe developmental delay and epileptic encephalopathy, with generalized tonic–clonic and myoclonic seizures being the most common. Seizure onset typically occurred within the first 2 years of life. Notably, disease severity correlated with the location of the variant within the YWHAG gene; mutations within the highly conserved triad domain, particularly Arg132 and Tyr133, were associated with more severe phenotypes, whereas variants outside this region tended to produce milder clinical outcomes (Tan et al., 2025).

Taken together, these findings suggest emerging genotype–phenotype correlations in YWHAG-related disorders. Variants located within the Arg132-Arg57-Tyr133 triad, especially Arg132His, Arg132Cys, Arg132Gly, Arg57Cys, and Tyr133Ser, are frequently associated with severe DEE phenotypes, such as early-onset seizures, poor AED response, and significant Developmental Delay or intellectual disability. In contrast, truncating mutations like Arg42Ter or variants outside the triad, like Lys50Gln, Ser180Tyr, Ala193Val, and Glu207Lys are more likely to present with milder clinical features such as febrile seizures or ASD. Novel mutations such as Asn212_Ser215del and Arg125His further expand the genetic and phenotypic landscape of this disorder (Amato et al., 2024). Differences in AED responsiveness, including favorable responses to VPA, ethosuximide, or combination therapies, may guide personalized treatment strategies based on the specific mutation.

### 3.4 *De novo* YWHAG mutations

Of the 25 reported YWHAG variants listed in Table 2, 18 have been confirmed as *de novo*, three as inherited (Arg42Ter, Thr31Aspfs5*, Ile63Thrfs3*), and two variants (Arg57His and Arg132Cys) have been reported as occurring either *de novo* or inherited, depending on the case. Inheritance status remains unknown for two variants (Arg125His and Asn212_Ser215del) due to lack of parental testing or published data (Guella et al., 2017; Ye et al., 2021; Amato et al., 2024; Tan et al., 2025). The exact clinical proportion of *de novo* versus inherited YWHAG mutation cases is unknown, although recent cohort data suggest that the vast majority, up to 86.7%, are *de novo*, as reported in a study of 15 individuals with YWHAG-related epilepsy (Tan et al., 2025). *De novo* mutations originate during gametogenesis or early embryonic development. They are absent from the DNA of either parent and instead emerge either in one of the germ line cells (sperm or egg) or in a somatic cell immediately after fertilization. Consequently, affected individuals carry one healthy copy of the gene and one mutated variant. If the mutation arises in the parental germ line or early in embryogenesis, it typically affects all cells. Mutations occurring later in development may be confined to specific tissues (Tian et al., 2021). While the YWHAG mutations responsible for neurological disorders are predominantly considered germline events, further research is required to distinguish between germline and somatic mutations in this context.

**Table 2:** Characteristics of YWHAG mutations.

YWHAG variant	Inheritance	Clinical phenotype	AED response	References
Missense mutations				
Glu15Ala	*De novo*	EE, GE, FS, SE, Hypotonia,	VPA DVP	Guella et al. (2017), Ye et al. (2021), Yi et al. (2022), and Tan et al. (2025)
Lys50Gln	*De novo*	ASD	Unknown	Guella et al. (2017) and Ye et al. (2021)
Arg57Cys	*De novo*	DEE, MS, FLE, Focal, GTCS, MAE, MA, Abs, Atyp Abs, ES, Ataxia, Tremor, Clumsiness, ASD, ADHD	VPA, ACTH, CBZ, CLB, ESM, LEV, LTG, OXC, TPM	Kanani et al. (2020), Ye et al. (2021), Yi et al. (2022), Cetica et al. (2024), and Tan et al. (2025)
Arg57Gly	*De novo*	DEE, MAE, GTCS, Focal, MA, Atyp Abs, Abs, ASD	CLB, ESM, LEV	Kanani et al. (2020), Ye et al. (2021), Yi et al. (2022), and Tan et al. (2025)
Arg57His	*De novo* or Inherited	DEE, GTCS, FS, Afeb, Atyp Abs, Abs, Ataxia, Clumsiness, ASD, ADHD	VPA, CBZ, CBD, CLB, ESM, LTG, STP	Sedláčková et al. (2021), Yi et al. (2022), Cetica et al. (2024), and Tan et al. (2025)
Arg125His	Unknown	FS, Abs	Unknown	Amato et al. (2024)
Lys125Glu	*De novo*	GE, FS, Afeb, MS	VPA	Ye et al. (2021) and Tan et al. (2025)
Asp129Glu	*De novo*	DEE, MS, MAE, MA, LGS, GTCS, Atyp Abs, Ataxia, Tremor	VPA, CBZ, CLZ, LEV, LTG, PYR, TPM	Epi4K Consortium and Epilepsy Phenome/Genome Project (2013), Guella et al. (2017), Ye et al. (2021), and Tan et al. (2025)
Arg132Cys	*De novo* or inherited	DEE, GTCS, MS, MAE, MA, Focal, Abs, Atyp Abs, ES, Ataxia, Tremor, Hypotonia, Clumsiness, ASD, ADHD, GE	VPA, BVC, CBD, CBZ, CLB, CLZ, DVP, ESM, LCM, LEV, LTG, PB, PMP, STP, TPM	Guella et al. (2017), Minardi et al. (2020), Kanani et al. (2020), Ye et al. (2021), Yi et al. (2022), Iodice et al. (2022), Cetica et al. (2024), and Tan et al. (2025)
Arg132Gly	*De novo*	DEE, MAE, GTCS, MS, MA, Ataxia, Clumsiness, Tremor, ASD	VPA, CLZ, ESM, LEV	Cetica et al. (2024) and Tan et al. (2025)
Arg132His	*De novo*	DEE, GTCS, MS, MAE, MA, Abs, Atyp Abs, Ataxia, Clumsiness, Tremor, ASD, ADHD	VPA, CBD, CBZ, CLB, CLZ, ESM, HC, LEV, STP, TPM	Brunet et al. (2021), Cetica et al. (2024), and Tan et al. (2025)
Tyr133Asn	*De novo*	GTCS	LEV	Tan et al. (2025)
Tyr133Cys	*De novo*	DEE, IESS, ES	ACTH, VGB	Tan et al. (2025)
Tyr133Ser	*De novo*	DEE, GTCS	Unknown	Guella et al. (2017), Kanani et al. (2020), Ye et al. (2021), and Tan et al. (2025)
Leu177Ile	*De novo*	EE	Unknown	Kanani et al. (2020), Ye et al. (2021), Yi et al. (2022), and Tan et al. (2025)
Asn178Asp	*De novo*	EE, Atyp Abs, ASD	ESM, LTG	Kanani et al. (2020), Ye et al. (2021), and Tan et al. (2025)
Ser180Tyr	*De novo*	MEI, MS, GTCS, Ataxia	VPA, LEV, TPM	Yi et al. (2022) and Tan et al. (2025)
Ala193Val	*De novo*	DEE, GTCS, Abs	VPA	Cetica et al. (2024) and Tan et al. (2025)
Glu207Lys	*De novo*	DEE, GTCS, MS, Abs	VPA, CBZ, LEV, ZNS	Stern et al. (2021), Yi et al. (2022), Cetica et al. (2024), and Tan et al. (2025)
Frameshift mutations				
Thr31Aspfs*5	Inherited	GE, GTCS, ASD	VPA, LEV	Cetica et al. (2024) and Tan et al. (2025)
Ile63Thrfs*3	Inherited	GE, Abs, ADHD	VPA, ESM, LTG	Cetica et al. (2024) and Tan et al. (2025)
Ser102Alafs*7	*De novo*	GE, Abs, Focal, MS, ADHD	VPA, LTG	Iodice et al. (2022), Amato et al. (2024), and Tan et al. (2025)
Nonsense truncating mutations				
Arg42Ter	Inherited	GE, GTCS, FS, Afeb, MS	VPA	Ye et al. (2021), Iodice et al. (2022), and Tan et al. (2025)
Trp233Ter	*De novo*	GE	VPA, OXC	Cetica et al. (2024) and Tan et al. (2025)
In-frame deletion mutations				
Asn212_Ser215del	Unknown	SE, DE, GTCS, Dyskinesia, ASD	VPA, CLZ	Amato et al. (2024)

ADHD, Attention Deficit/Hyperactivity Disorder; Afeb, Afebrile Seizures; ASD, Autism Spectrum Disorder; Atyp Abs, Atypical Absences; Abs, Absence Seizures; DE, Developmental Encephalopathy; DEE, Developmental and Epileptic Encephalopathy; EE, Epileptic Encephalopathy; ES, Epileptic Spasms; FLE, Frontal Lobe Epilepsy; Focal, Focal Seizures; FS, Febrile Seizures; GE, Generalized Epilepsy; GTCS, Generalized Tonic–Clonic Seizures; ID, Intellectual Disability; IESS, Infantile Epileptic Spasms Syndrome; LGS, Lennox–Gastaut Syndrome; MA, Myoclonic Absences; MAE, Myoclonic-Atonic Epilepsy; MEI, Myoclonic Epilepsy of Infancy; MS, Myoclonic Seizures; SE, Status Epilepticus; ACTH, Adrenocorticotropic Hormone; BVC, Brivaracetam; CBD, Cannabidiol; CBZ, Carbamazepine; CLB, Clobazam; CLZ, Clonazepam; DVP, Divalproex Sodium; ESM, Ethosuximide; HC, Hydrocortisone; LCM, Lacosamide; LEV, Levetiracetam; LTG, Lamotrigine; OXC, Oxcarbazepine; PB, Phenobarbital; PMP, Perampanel; PYR, Pyridoxine; STP, Stiripentol; TPM, Topiramate; VGB, Vigabatrin; VPA, Valproic Acid; ZNS, Zonisamide.

Despite being spontaneous, *de novo* mutations can have hereditary implications. If the mutation occurs in the germline, it can be passed on to future generations, becoming part of the individual’s genetic legacy. In the case of YWHAG mutations, these genetic alterations are generally assumed to occur during gametogenesis, making them germline in nature (Acuna-Hidalgo et al., 2015). However, the possibility of somatic mutations remains, depending on the timing and location of the mutation. Documented YWHAG variants exhibit a spectrum of impacts on individuals, with severity ranging widely. These mutations highlight the complexity of YWHAG’s role in brain function and emphasize the need for further research to improve diagnostic and therapeutic approaches for YWHAG-related conditions.

## 4 Potential mechanisms of mutated 14-3-3γ in DEE

### 4.1 Potential cause of EE and DE at different levels of nervous system

Epileptic encephalopathy (EE) and developmental encephalopathy (DE) may arise from distinct disruptions in brain function at multiple levels: neuronal, synaptic, and network (Table 3). The 14-3-3γ protein, plays a significant role in neuronal signaling, synaptic function, and neurodevelopment. Mutations in the YWHAG gene disrupt the structure and function of 14-3-3γ, potentially contributing to the pathogenesis of DEE by altering these processes (Komoike et al., 2010; Kim et al., 2019).

**Table 3:** Seizure origins at different levels of nervous system and associated dysfunctions at each level.

Level within nervous system where seizure originates:	Potential underlying impairments or causes at each level:
Neuron/Cellular	Dysfunctional ion channels; neuronal hyperexcitability.
Synapse	Imbalance of excitatory vs. inhibitory neurotransmitters.
Network/Circuit	Atypical synchronization patterns; dysregulated gene expression; disordered neuronal morphology and lamination.

At the neuronal level, 14-3-3γ is involved in regulating ion channels that maintain neuronal excitability. Mutated 14-3-3γ could result in impaired ion channel function, which may lead to neuronal hyperexcitability and increase the likelihood of spontaneous action potentials and seizure activity (Roy et al., 2021; Logue et al., 2024). Disrupted ion channel regulation may also interfere with neuronal maturation and synaptic development, contributing to the developmental delays (Komoike et al., 2010; Foote et al., 2015).

At the synaptic level, 14-3-3γ modulates synaptic plasticity, neurotransmitter release, and receptor trafficking. Mutated 14-3-3γ could lead to dysregulated glutamate and GABA signaling, disrupting the balance between excitatory and inhibitory signals (Qiao et al., 2014; Wen et al., 2022). This disruption may promote epileptiform activity and interfere with synaptic pruning, contributing to abnormal neural circuit formation (Guella et al., 2017; Feng et al., 2022). Such alterations in synaptic function are linked to cognitive deficits and learning and memory impairments (Foote et al., 2015).

At the network level, 14-3-3γ regulates neuronal differentiation, migration, and circuit activity. Mutations in 14-3-3γ can disrupt network synchronization, leading to abnormal firing patterns that promote seizure propagation (Cornell et al., 2016; Roy et al., 2021). Impaired long-range connectivity may further affect cognition, motor coordination, and sensory processing, contributing to the neurodevelopmental deficits (Guella et al., 2017). Cortical disorganization from 14-3-3γ dysfunction may contribute to developmental delays and cognitive impairments (Sehgal et al., 2014; Wachi et al., 2016).

The following sections of this paper will further elaborate on the impact of YWHAG mutation at the neuronal level by examining intrinsic excitability ion channel dysfunction, at the synaptic level by analyzing synaptic transmission and plasticity, and at the network level by investigating circuit connectivity and neuronal migration.

### 4.2 Neuronal hyperexcitability in 14-3-3 FKO mouse model

**Figure 7:**
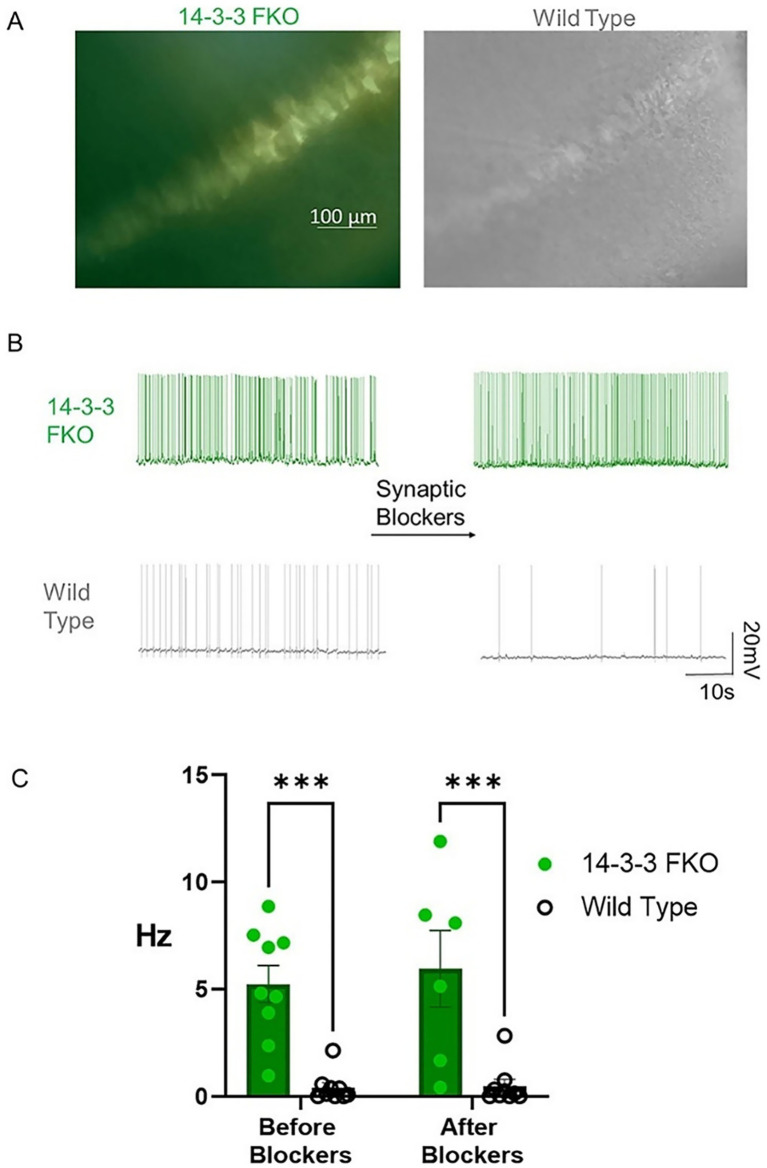
14-3-3 FKO hippocampal CA1 neurons fire more APs than WT neurons in the presence and absence of synaptic blockers. **(A)** Hippocampal slice images captured using phase contrast and fluorescence microscopy show 14-3-3 FKO neurons (left) identified by their YFP fluorescence, indicating difopein expression. **(B)** Traces of spontaneous AP firing in 14-3-3 FKO and WT neurons under whole-cell configuration, before and after synaptic blocker application. **(C)** Group data showing a higher AP firing rate for 14-3-3 FKO cells (*n* = 9 before blockers, 6 after blockers) than WT cells (*n* = 9 before blockers, 8 after blockers). AP, Action Potential; FKO, Functional Knockout; WT, Wildtype; CA1, one of four hippocampal subfields that make up hippocampus structure. Source: Figure 1 from Logue et al. (2024).

To investigate the functions of 14-3-3 protein family in the nervous system, our lab developed the 14-3-3 functional knockout (FKO) mouse model. These mice express YFP-fused difopein, a dimeric fourteen-three-three (14-3-3) peptide inhibitor that disrupts 14-3-3 interactions by antagonizing it is binding with endogenous partners, under the neuron-specific Thy-1 promoter (Qiao et al., 2014). Difopein-expressing neurons appear fluorescent under microscopy, distinguishing them from wild-type (WT) neurons (Figure 7A). Since difopein inhibits all 14-3-3 isoforms, this model provides a tool to study how 14-3-3 proteins contribute to neuronal excitability, synaptic function, and behavior.

Neuronal hyperexcitability, defined by excessive action potential firing, is a defining characteristic of EE. The 14-3-3 proteins modulate cellular excitability, and loss of 14-3-3 leads to impacts intrinsic excitability (Foote and Zhou, 2012). In the FKO mouse model, electrophysiological recordings confirmed persistently higher spontaneous action potential firing in hippocampal CA1 pyramidal FKO neurons compared to hippocampal WT neurons; this increased excitability persisted even in the presence of excitatory and inhibitory synaptic blockers, indicating that the hyperexcitability arises from intrinsic cellular mechanisms rather than synaptic input (Figures 7B,C) (Logue et al., 2024).

In a different mouse model, Ywhag knockdown in the anterodorsal (AD) thalamus induced neuronal hyperexcitability, decreased the action potential threshold and abnormal firing activity; chemogenetic normalization of excitability restored physiological firing patterns, highlighting the link between 14-3-3γ dysfunction and neuronal excitability (Roy et al., 2021).

While the FKO model demonstrates that pan-14-3-3 inhibition can cause neuronal hyperexcitability, it does not isolate the specific contribution of 14-3-3γ or replicate the effect of specific missense mutations. The difopein construct disrupts all isoforms of 14-3-3, limiting its ability to model isoform-specific DEE mechanisms or genotype–phenotype relationships observed in DEE56. To model the effects of a missense mutation more precisely, efforts have been made to generate isogenic iPSC-derived neural cultures engineered via CRISPR to carry the Arg132Cys mutation. This model is being used in our lab to assess intrinsic excitability in 2D cortical neurons and network activity in 3D forebrain organoids. This approach enables direct comparison between the functional impact of the Arg132Cys variant and complete 14-3-3γ loss, while also allowing investigation of cell-type–specific effects relevant to YWHAG-associated pathology.

### 4.3 14-3-3γ deficiency in ion channel dysregulation

Neuronal hyperexcitability in the 14-3-3 FKO mouse model is linked to disruptions in specific ion channels, such as voltage-gated calcium channels (VGCCs), NMDA receptors (NMDARs), and inward-rectifying potassium channel (KIR2.2). A rightward shift in calcium currents of VGCCs (Logue et al., 2024), downregulation of NMDARs, (Qiao et al., 2014), and a reduction in KIR2.2 currents (Roy et al., 2021) are all significant alterations observed in the 14-3-3 FKO mouse model, contributing to neural hyperexcitability and disrupted burst-firing patterns. These ion channel modifications have been linked to the altered synaptic plasticity and behavioral deficits observed in FKO mice (Zhang et al., 2022).

14-3-3 proteins, including the 14-3-3γ isoform, regulate ion channels through phosphorylation-dependent binding, modulating channel gating, conformation, and trafficking. Many ion channels contain phosphorylation sites that enable 14-3-3 interactions, influencing their localization and function. For example, 14-3-3γ stabilizes KIR2.2 expression and activity, and its loss in Ywhag knockdown mice leads to decreased KIR2.2 currents, which impairs potassium frow and leads to membrane depolarization, thereby increasing neuronal excitability (Roy et al., 2021). These changes may contribute to EE, where hyperexcitability predisposes neurons to pathological firing patterns. Additionally, chemogenetic targeted viral restoration of ion channel function in a Ywhag knockdown model reversed neuronal hyperexcitability (Roy et al., 2021).

14-3-3γ likely regulates additional ion channels involved in neuronal excitability. For example, VGCC disruption affects calcium-activated potassium channels by reducing calcium influx, impairing their activation and leading to deficient potassium outflow during afterhyperpolarization, which results in increased neuronal excitability in CA1 pyramidal cells (Lancaster and Adams, 1986). 14-3-3γ also interacts with numerous other intracellular proteins, including 170 identified 14-3-3-associated targets that regulate ion channel activity and receptor trafficking (Jin et al., 2004). Separate studies have also identified the role of 14-3-3 proteins in transmembrane receptor trafficking (Cho and Park, 2020).

Additionally, studies show that 14-3-3γ enhances the surface expression of ANO1, a calcium-activated chloride channel; silencing ANO1 inhibits glioblastoma cell migration and invasion (Lee et al., 2016). In hippocampal astrocytes, 14-3-3γ exclusively binds to Best1, a calcium-activated anion channel, enhancing its surface expression and promoting glutamate release; silencing 14-3-3γ reduces Best1 expression (Oh et al., 2017). Additionally, 14-3-3γ interacts with TRPM4 channels, increasing their plasma membrane expression, whereas silencing reduces TRPM4 expression, affecting glutamate-induced cell death (Cho et al., 2014).

### 4.4 14-3-3γ regulation of excitatory and inhibitory neurotransmission in epilepsy

Glutamate, the primary excitatory neurotransmitter, activates NMDARs, mediating calcium influx and regulating neuronal excitability. Excessive glutamate overstimulates NMDARs, leading to hyperexcitability and seizures. Abnormal glutamate signaling is a key factor in epilepsy pathophysiology, with elevated extracellular glutamate levels commonly observed in both animal models and human patients, especially in cases of temporal lobe epilepsy (Guella et al., 2017). Chronic glutamate excess alters synaptic plasticity and network function, particularly in hippocampal circuits involved in temporal lobe epilepsy (Barker-Haliski and White, 2015). Increased extracellular glutamate in the brain, as well as a reduction in GABA concentrations, can result in excitotoxicity, seizures, and cell death (Sarlo and Holton, 2021).

14-3-3 proteins, including 14-3-3γ, regulate NMDAR function. In 14-3-3 FKO mice, reduced synaptic NMDAR expression correlates with impairments in associative learning, memory, and synaptic plasticity within hippocampus (Qiao et al., 2014). Behavioral assessments, such as Y-maze spontaneous alternation, further confirm deficits in spatial working memory in FKO mice (Foote et al., 2015). Molecular studies demonstrate NMDAR hypofunctionality, including reduced GluN1 and GluN2A levels in hippocampal postsynaptic densities, a decreased NMDA/AMPA receptor ratio, and diminished NMDAR-mediated excitatory postsynaptic currents in hippocampal neurons (Qiao et al., 2014). *In vitro*, inhibiting 14-3-3 in with difopein in primary glutamatergic cortical and hippocampal neurons disrupts NMDAR localization and reduces surface expression of GluN1, GluN2A, and GluN2B subunits, thereby highlighting 14-3-3 role in receptor assembly, trafficking, and postsynaptic membrane insertion (Lee et al., 2021).

The YWHAG gene and 14-3-3γ protein are implicated in epilepsy-associated pathways, potentially interacting with glutamate metabolism genes (Epi4K Consortium and Epilepsy Phenome/Genome Project, 2013). Mutations in key regulators of glutamate signaling, SLC1A2, GRIN2A, and GRIN2B are linked to onset of EE (Feng et al., 2022). Although 14-3-3γ’s precise role in these pathways requires further study, its involvement in protein–protein interactions and signaling cascades suggests indirect contributions.

Additionally, GABA, the primary inhibitory neurotransmitter, activates GABA type A receptors (GABAARs), mediating chloride influx and reducing neuronal excitability. Reduced GABA signaling weakens inhibition, increasing hyperexcitability and seizure risk. A study demonstrated that in rats, 14-3-3 proteins stabilize interactions with HAP1, which regulates GABAAR surface expression and inhibitory synaptic transmission; disrupting the 14-3-3γ/HAP1 complex weakens GABAAR signaling, thereby increasing neuronal excitability and seizure susceptibility (Wen et al., 2022).

### 4.5 14-3-3γ dysregulation in neuronal migration and epilepsy

Neuronal migration is essential for proper brain development, and disruptions in 14-3-3γ function can impair this process, contributing to cortical malformations associated with epilepsy (Cornell and Toyo-oka, 2017). Loss of 14-3-3γ disrupts cell–cell adhesion and trafficking of plakoglobin, a desmosomal protein, to the cell border, therefore compromising neuronal positioning and connectivity (Sehgal et al., 2014). 14-3-3γ also interacts with cytoplasmic linker proteins (CLASPs) to regulate cytoskeletal dynamics, including microtubules and actin filament organization, which are critical for neuronal migration (Jin et al., 2004). These interactions are essential for guiding neurons to their target locations, and any imbalance in 14-3-3γ levels can disrupt normal migration. Dysregulation of 14-3-3γ can prevent neurons from reaching their proper locations, impairing cortical connectivity and leading to abnormal network synchronization, factors closely associated with epilepsy (Guella et al., 2017). Neuronal migration during cortical development is modulated by the paracrine actions of glutamate and GABA neurotransmitters, which influence balance of excitatory and inhibitory signals (Luhmann et al., 2015); the imbalance can contribute to the onset of EE.

Any deviations in 14-3-3γ expression, whether through depletion or overexpression, can impair neuronal positioning and contribute to cortical malformations. Mouse models with decreased levels of 14-3-3γ due to genetic ablation cause delayed migration of pyramidal neurons in the cerebral cortex (Wachi et al., 2016). Conversely, overexpression of 14-3-3γ similarly disrupts migration patterns (Cornell et al., 2016). Thusly, that both reduced and excessive 14-3-3γ expression impair pyramidal neuron migration, highlighting the need for balanced 14-3-3γ expression to maintain proper cortical development and organization. Loss of 14-3-3γ also delays pyramidal neuron migration in the cerebral cortex, highlighting its role in cortical development (Mizuno et al., 2007).

Loss of 14-3-3γ contributes to neuronal migration deficits (Wachi et al., 2016) and to network imbalances that exacerbate epilepsy severity (Guella et al., 2017). 14-3-3γ dysregulation is implicated in epilepsy, from mild forms such as ME and FS to severe cases like EE (Komoike et al., 2010). In rat models, acute seizures induced by kainic acid reduce hippocampal 14-3-3γ levels; however, no significant 14-3-3γ alterations have been observed in the hippocampus of human patients with chronic epilepsy, suggesting differences between acute and chronic stages or species-specific variations (Schindler et al., 2006).

Recent studies have demonstrated that homozygous knockout of 14-3-3γ in mice results in lethality before postnatal day 21, underscoring its essential role in early neurodevelopment (Cho et al., 2023). Heterozygous Ywhag knockout leads to motor coordination deficits and altered dopaminergic signaling, further supporting the involvement of 14-3-3γ in critical developmental pathways (Cho et al., 2023). Although the precise mechanisms underlying the lethality remain unclear, these findings suggest that 14-3-3γ is required not only for neuronal migration but also for broader processes such as cortical organization, synaptic maturation, and survival signaling during early brain development.

### 4.6 Behavioral and developmental impacts of 14-3-3γ dysregulation

Studies on 14-3-3 FKO mice have shown significant behavioral deficits, including social withdrawal, impaired associative learning and memory, and novelty-induced hyperlocomotion—symptoms resembling schizophrenia (Foote et al., 2015). These deficits are linked to altered neurotransmission, reduced dendritic complexity, and decreased spine density in forebrain excitatory neurons. Our lab has demonstrated that these structural changes likely result from impaired 14-3-3 regulation of phosphorylated cofilin, a key protein in actin cytoskeletal dynamics (Foote et al., 2015).

In Ywhag knockdown mouse model, 14-3-3γ deficiency resulted in neuronal hyperexcitability and impaired contextual fear conditioning memory, thereby linking between 14-3-3γ dysfunction and cognitive deficits; however, a chemogenetic viral approach to normalize neuronal excitability resulted in behavioral restoration (Roy et al., 2021). Mutations or dysregulation of the YWHAG gene disrupt essential developmental processes. Homozygous 14-3-3γ knockout mice are prenatally lethal, while heterozygous mice exhibit developmental delays, hyperactivity, anxiety-like and depressive-like behaviors, and heightened stress sensitivity (Kim et al., 2019). Zebrafish lacking 14-3-3γ show delayed brain development and reduced brain size, underscoring its importance in early neural formation (Komoike et al., 2010).

### 4.7 14-3-3γ dysregulation in neurodevelopmental, psychiatric, and neurodegenerative disorders

Expression and role of 14-3-3γ varies across development, aging, and brain regions, with potential implications for neurodevelopmental and neurodegenerative disorders. In individuals with Down syndrome, 14-3-3γ levels were reduced in the fetal cortex (Peyrl et al., 2002) but elevated in elderly individuals (Fountoulakis et al., 1999). In individuals with Alzheimer’s Disease, 14-3-3γ levels were elevated in the cortex overall (Fountoulakis et al., 1999) but specifically decreased in the frontal cortex (Gu et al., 2020).

Dysregulation of 14-3-3γ is associated with multiple neurodevelopmental disorders. Loss of 14-3-3γ function disrupts cortical development and connectivity, contributing to cognitive deficits in Autism Spectrum Disorder (ASD) (Guella et al., 2017). Chromosomal abnormalities, such as deletion or duplication at the 7q11.23 locus, where the ywhag gene is located in mice, are associated with Williams-Beuren syndrome—a disorder marked by developmental delays, intellectual disabilities, and epilepsy (Kim et al., 2019). Additionally, our lab has shown that 14-3-3 inhibition in the brain leads to NMDAR deficits, which may contribute to schizophrenia-related synaptic alterations and behavioral abnormalities (Foote et al., 2015).

14-3-3γ is also implicated in neurodegenerative diseases. Increased 14-3-3γ expression correlates with cognitive decline, including dementia, and motor dysfunction such as muscle stiffness and involuntary movements in Creutzfeldt-Jakob disease (Wiltfang et al., 1999). In Parkinson’s Disease (PD), aged 14-3-3γ-deficient heterozygous knockout mice exhibit reduced dopamine levels, altered dopamine metabolism, changes in protein phosphorylation, and PD-like symptoms, including impaired motor coordination and nest-building deficits, suggesting a role for 14-3-3γ in PD pathophysiology (Cho et al., 2023).

## 5 Conclusion

The 14-3-3γ protein, encoded by the YWHAG gene, plays an important role in maintaining neuronal function and homeostasis (Foote and Zhou, 2012; Cho and Park, 2020). Clinical evidence suggests mutated 14-3-3γ diminishes its functionality, potentially leading to pathogenesis of DEE, a severe neurodevelopmental disorder characterized by early-onset epilepsy, cognitive impairments, and developmental delays (Guella et al., 2017; Kanani et al., 2020). Mutations in YWHAG, particularly *de novo* missense variants, disrupt the regulatory functions of 14-3-3γ, leading to diverse epilepsy phenotypes and significant neurodevelopmental challenges (Ye et al., 2021; Gheorghita et al., 2023; Yi et al., 2022).

The 14-3-3γ exerts its functions through phosphorylation-dependent interactions with target proteins, regulating their stability, activity, and localization (Obsil and Obsilova, 2011; Gogl et al., 2021). These interactions are required for neuronal excitability, synaptic plasticity, and cortical development (Qiao et al., 2014; Foote et al., 2015). Mutations, such as the well-characterized Arg132Cys variant, impair 14-3-3γ’s binding groove, destabilizing its molecular partners, and disrupting ion channel regulation, NMDA receptor trafficking, and cytoskeletal dynamics (Valente et al., 2012; Skjevik et al., 2014). These disruptions often result in neural hyperexcitability, impaired neuronal migration, and cortical malformations, aligning with the clinical manifestations observed in YWHAG mutation carriers (Wachi et al., 2016; Guella et al., 2017).

Animal and cellular models provide compelling insights into the role of 14-3-3γ in neurodevelopment. FKO mice reveal hyperexcitability and altered firing patterns due to dysregulated ion channels, such as KIR2.2 and calcium-activated potassium channels (Roy et al., 2021; Logue et al., 2024). Additionally, these models demonstrate deficits in synaptic plasticity, reduced NMDA/AMPA receptor ratios, and diminished excitatory postsynaptic currents (Qiao et al., 2014; Lee et al., 2021). Behavioral studies in FKO mice further emphasize the translational relevance of 14-3-3γ dysfunction, showcasing phenotypes resembling human neurodevelopmental and psychiatric disorders, such as social withdrawal, hyperlocomotion, and cognitive impairments (Foote et al., 2015; Kim et al., 2019).

The clinical variability of YWHAG-related DEE is influenced by the nature and location of mutations. Missense mutations affecting the conserved Arg132-Arg57-Tyr133 triad result in severe dysfunction (Skjevik et al., 2014; Ye et al., 2021), whereas truncating mutations produce milder phenotypes due to haploinsufficiency (Kanani et al., 2020). These genotype–phenotype correlations provide valuable insights into the molecular mechanisms of DEE56, indicating that 14-3-3γ maintains a complex role in neurological disorders.

Despite significant progress, gaps remain in understanding molecular mechanisms by which YWHAG mutations contribute to DEE. Current therapeutic approaches, such as anticonvulsants like valproate, provide symptomatic relief but fail to address the underlying pathology (Kanani et al., 2020). Future research should clarify the molecular interactions and signaling pathways regulated by 14-3-3γ in DEE. Improved modeling using patient-derived organoids and advanced electrophysiological tools will help identify how YWHAG mutations disrupt neuronal function.

Preclinical studies have shown that chemogenetic restoration of excitability can reverse behavioral and electrophysiological deficits caused by 14-3-3γ dysfunction (Roy et al., 2021), supporting the potential of targeted therapies such as small-molecule modulators or gene-editing strategies. Pathogenic YWHAG mutations also impair 14-3-3γ localization and phosphotarget binding, and a recent high-throughput screen identified nafamostat as a potential small-molecule compound to partially restore these interactions (Larasati et al., 2025), supporting the therapeutic potential of pharmacological rescue strategies for 14-3-3γ dysfunction.

Recent advances in small-molecule drug discovery have expanded the therapeutic potential of targeting 14-3-3 protein–protein interactions (PPIs). These modulators fall into two main categories: inhibitors, which disrupt pathological 14-3-3 interactions, and stabilizers, which enhance weakened interactions, such as those caused by YWHAG mutations that impair phosphotarget binding. Stabilizers like Epibestatin, Pyrrolidone1, and compound 21 have been identified through high-throughput screening, in silico docking, and structure-based drug design, and show efficacy in restoring 14-3-3/client engagement in various biological contexts (Stevers et al., 2018).

Another study employed a structure-guided fragment-linking approach to generate molecular glues that reinforce 14-3-3 interactions with phosphopeptides (Visser et al., 2023). Although their initial proof-of-concept targeted an estrogen receptor–derived peptide, the binding interface of the highly conserved 14-3-3 phosphopeptide groove, is shared across all isoforms, including 14-3-3γ. This strategy presents a blueprint for designing small molecules capable of stabilizing disrupted interactions caused by pathogenic YWHAG variants.

Continued investigation of 14-3-3γ expression across developmental stages, brain regions, and disease states will be essential to refine our understanding of its role in neuronal health and DEE pathogenesis and to advance precision therapies for YWHAG-related DEE.

## References

[ref1] Abdrabou A. Brandwein D. Wang Z. (2020). Differential subcellular distribution and translocation of seven 14-3-3 isoforms in response to EGF and during the cell cycle. Int. J. Mol. Sci. 21:318. doi: 10.3390/ijms21010318, PMID: 31906564 PMC6981507

[ref2] Acuna-Hidalgo R. Bo T. Kwint M. P. van de Vorst M. Pinelli M. Veltman J. A.. (2015). Post-zygotic point mutations are an Underrecognized source of *De novo* genomic variation. Am. J. Hum. Genet. 97, 67–74. doi: 10.1016/j.ajhg.2015.05.008, PMID: 26054435 PMC4571017

[ref3] Aitken A. Howell S. Jones D. Madrazo J. Patel Y. (1995). 14-3-3 α and δ are the phosphorylated forms of Raf-activating 14-3-3 β and ζ. J. Biol. Chem. 270, 5706–5709. doi: 10.1074/jbc.270.11.5706, PMID: 7890696

[ref4] Amato M. E. Balsells S. Martorell L. Alcalá San Martín A. Ansell K. Børresen M. L.. (2024). Developmental and epileptic encephalopathy 56 due to YWHAG variants: 12 new cases and review of the literature. Eur. J. Paediatr. Neurol. 53, 63–72. doi: 10.1016/j.ejpn.2024.10.005, PMID: 39413657

[ref5] Barker-Haliski M. White H. S. (2015). Glutamatergic mechanisms associated with seizures and epilepsy. Cold Spring Harb. Perspect. Med. 5:a022863. doi: 10.1101/cshperspect.a022863, PMID: 26101204 PMC4526718

[ref6] Brunet T. Jech R. Brugger M. Kovacs R. Alhaddad B. Leszinski G.. (2021). *De novo* variants in neurodevelopmental disorders—experiences from a tertiary care center. Clin. Genet. 100, 14–28. doi: 10.1111/cge.13946, PMID: 33619735

[ref7] Cetica V. Pisano T. Lesca G. Marafi D. Licchetta L. Riccardi F.. (2024). Clinical and molecular characterization of patients with *YWHAG*-related epilepsy. Epilepsia 65, 1439–1450. doi: 10.1111/epi.17939, PMID: 38491959

[ref8] Chaudhri M. Scarabel M. Aitken A. (2003). Mammalian and yeast 14-3-3 isoforms form distinct patterns of dimers in vivo. Biochem. Biophys. Res. Commun. 300, 679–685. doi: 10.1016/S0006-291X(02)02902-9, PMID: 12507503

[ref9] Chen X. Q. Chen J. G. Zhang Y. Hsiao W. W. L. Yu A. C. H. (2003). 14-3-3γ is upregulated by in vitro ischemia and binds to protein kinase Raf in primary cultures of astrocytes. Glia 42, 315–324. doi: 10.1002/glia.10185, PMID: 12730952

[ref10] Chen X. Q. Fung Y.-W. W. Yu A. C. H. (2005). Association of 14-3-3γ and phosphorylated bad attenuates injury in ischemic astrocytes. J. Cereb. Blood Flow Metab. 25, 338–347. doi: 10.1038/sj.jcbfm.9600032, PMID: 15660102

[ref11] Cho C.-H. Kim E. Lee Y.-S. Yarishkin O. Yoo J. C. Park J.-Y.. (2014). Depletion of 14-3-3γ reduces the surface expression of transient receptor potential Melastatin 4b (TRPM4b) channels and attenuates TRPM4b-mediated glutamate-induced neuronal cell death. Mol. Brain 7:52. doi: 10.1186/s13041-014-0052-3, PMID: 25047048 PMC4115172

[ref12] Cho E. Park J. Hwang E. M. Kim H. W. Park J.-Y. (2023). 14-3-3γ haploinsufficiency leads to altered dopamine pathway and Parkinson’s disease-like motor incoordination in mice. Mol. Brain 16:2. doi: 10.1186/s13041-022-00990-z, PMID: 36604743 PMC9817279

[ref13] Cho E. Park J.-Y. (2020). Emerging roles of 14-3-3γ in the brain disorder. BMB Rep. 53, 500–511. doi: 10.5483/BMBRep.2020.53.10.158, PMID: 32958119 PMC7607152

[ref14] Coblitz B. Shikano S. Wu M. Gabelli S. B. Cockrell L. M. Spieker M.. (2005). C-terminal recognition by 14-3-3 proteins for surface expression of membrane receptors. J. Biol. Chem. 280, 36263–36272. doi: 10.1074/jbc.M507559200, PMID: 16123035

[ref15] Cornell B. Toyo-oka K. (2017). 14-3-3 proteins in brain development: neurogenesis, neuronal migration and Neuromorphogenesis. Front. Mol. Neurosci. 10:318. doi: 10.3389/fnmol.2017.00318, PMID: 29075177 PMC5643407

[ref16] Cornell B. Wachi T. Zhukarev V. Toyo-oka K. (2016). Overexpression of the 14-3-3gamma protein in embryonic mice results in neuronal migration delay in the developing cerebral cortex. Neurosci. Lett. 628, 40–46. doi: 10.1016/j.neulet.2016.06.009, PMID: 27288018

[ref17] Epi4K Consortium and Epilepsy Phenome/Genome Project (2013). *De novo* mutations in epileptic encephalopathies. Nature 501, 217–221. doi: 10.1038/nature12439, PMID: 23934111 PMC3773011

[ref18] Feng Y. Zhang C. Wei Z. Li G. Gan Y. Liu C.. (2022). Gene variations of glutamate metabolism pathway and epilepsy. Acta Epileptol. 4:31. doi: 10.1186/s42494-022-00103-2

[ref19] Ferl R. J. Manak M. S. Reyes M. F. (2002). The 14-3-3s. Genome Biol. 3:REVIEWS3010. doi: 10.1186/gb-2002-3-7-reviews3010, PMID: 12184815 PMC139387

[ref20] Foote M. Qiao H. Graham K. Wu Y. Zhou Y. (2015). Inhibition of 14-3-3 proteins leads to schizophrenia-related Behavioral phenotypes and synaptic defects in mice. Biol. Psychiatry 78, 386–395. doi: 10.1016/j.biopsych.2015.02.015, PMID: 25863357 PMC4544659

[ref21] Foote M. Zhou Y. (2012). 14-3-3 proteins in neurological disorders. Int J Biochem Mol Biol 3, 152–164, PMID: 22773956 PMC3388734

[ref22] Fountoulakis M. Cairns N. Lubec G. (1999). “Increased levels of 14-3-3 gamma and epsilon proteins in brain of patients with Alzheimer’s disease and down syndrome” in The molecular biology of down syndrome. ed. Lubec G. (Vienna: Springer Vienna), 323–335. 10.1007/978-3-7091-6380-1_23 10666687

[ref23] Gardino A. K. Smerdon S. J. Yaffe M. B. (2006). Structural determinants of 14-3-3 binding specificities and regulation of subcellular localization of 14-3-3-ligand complexes: a comparison of the X-ray crystal structures of all human 14-3-3 isoforms. Semin. Cancer Biol. 16, 173–182. doi: 10.1016/j.semcancer.2006.03.007, PMID: 16678437

[ref24] Gheorghita K. L. Ciurea A. V. Rizea R. E. (2023). A rare case of Yhwag gene mutation causing developmental and epileptic encephalopathy. Romanian Neurosurg. 22, 249–251. doi: 10.33962/roneuro-2023-045

[ref25] Gogl G. Tugaeva K. V. Eberling P. Kostmann C. Trave G. Sluchanko N. N. (2021). Hierarchized phosphotarget binding by the seven human 14-3-3 isoforms. Nat. Commun. 12:1677. doi: 10.1038/s41467-021-21908-8, PMID: 33723253 PMC7961048

[ref26] Gu Q. Cuevas E. Raymick J. Kanungo J. Sarkar S. (2020). Downregulation of 14-3-3 proteins in Alzheimer’s disease. Mol. Neurobiol. 57, 32–40. doi: 10.1007/s12035-019-01754-y, PMID: 31487003

[ref27] Guella I. McKenzie M. B. Evans D. M. Buerki S. E. Toyota E. B. Van Allen M. I.. (2017). *De novo* mutations in YWHAG cause early-onset epilepsy. Am. J. Hum. Genet. 101, 300–310. doi: 10.1016/j.ajhg.2017.07.004, PMID: 28777935 PMC5544417

[ref28] Guerrini R. Conti V. Mantegazza M. Balestrini S. Galanopoulou A. S. Benfenati F. (2023). Developmental and epileptic encephalopathies: from genetic heterogeneity to phenotypic continuum. Physiol. Rev. 103, 433–513. doi: 10.1152/physrev.00063.2021, PMID: 35951482 PMC9576177

[ref29] Huang X. Zheng Z. Wu Y. Gao M. Su Z. Huang Y. (2022). 14-3-3 proteins are potential regulators of liquid–liquid phase separation. Cell Biochem. Biophys. 80, 277–293. doi: 10.1007/s12013-022-01067-3, PMID: 35142991 PMC8830994

[ref30] Iodice A. Giannelli C. Soli F. Riva A. Striano P. (2022). Myoclonic epilepsy of infancy related to YWHAG gene mutation: towards a better phenotypic characterization. Seizure 94, 161–164. doi: 10.1016/j.seizure.2021.12.002, PMID: 34915349

[ref31] Jin J. Smith F. D. Stark C. Wells C. D. Fawcett J. P. Kulkarni S.. (2004). Proteomic, functional, and domain-based analysis of in vivo 14-3-3 binding proteins involved in cytoskeletal regulation and cellular organization. Curr. Biol. 14, 1436–1450. doi: 10.1016/j.cub.2004.07.051, PMID: 15324660

[ref32] Kanani F. Titheradge H. Cooper N. Elmslie F. Lees M. M. Juusola J.. (2020). Expanding the genotype–phenotype correlation of *de novo* heterozygous missense variants in *YWHAG* as a cause of developmental and epileptic encephalopathy. Am. J. Med. Genet. A 182, 713–720. doi: 10.1002/ajmg.a.61483, PMID: 31926053

[ref33] Kim D. E. Cho C.-H. Sim K. M. Kwon O. Hwang E. M. Kim H.-W.. (2019). 14-3-3γ Haploinsufficient mice display hyperactive and stress-sensitive Behaviors. Exp. Neurobiol. 28, 43–53. doi: 10.5607/en.2019.28.1.43, PMID: 30853823 PMC6401549

[ref34] Komoike Y. Fujii K. Nishimura A. Hiraki Y. Hayashidani M. Shimojima K.. (2010). Zebrafish gene knockdowns imply roles for human *YWHAG* in infantile spasms and cardiomegaly. Genesis 48, 233–243. doi: 10.1002/dvg.20607, PMID: 20146355

[ref35] Lancaster B. Adams P. R. (1986). Calcium-dependent current generating the afterhyperpolarization of hippocampal neurons. J. Neurophysiol. 55, 1268–1282. doi: 10.1152/jn.1986.55.6.1268, PMID: 2426421

[ref36] Larasati Y. A. Solis G. P. Koval A. Korff C. Katanaev V. L. (2025). A personalized 14-3-3 disease-targeting workflow yields repositioning drug candidates. Cells 14:559. doi: 10.3390/cells14080559, PMID: 40277885 PMC12025923

[ref37] Lee G. S. Zhang J. Wu Y. Zhou Y. (2021). 14-3-3 proteins promote synaptic localization of N-methyl d-aspartate receptors (NMDARs) in mouse hippocampal and cortical neurons. PLoS One 16:e0261791. doi: 10.1371/journal.pone.0261791, PMID: 34962957 PMC8714094

[ref38] Lee Y.-S. Lee J. K. Bae Y. Lee B.-S. Kim E. Cho C.-H.. (2016). Suppression of 14-3-3γ-mediated surface expression of ANO1 inhibits cancer progression of glioblastoma cells. Sci. Rep. 6:26413. doi: 10.1038/srep26413, PMID: 27212225 PMC4876403

[ref39] Logue J. B. Vilmont V. Zhang J. Wu Y. Zhou Y. (2024). Inhibition of 14-3-3 proteins increases the intrinsic excitability of mouse hippocampal CA1 pyramidal neurons. Eur. J. Neurosci. 59, 3309–3321. doi: 10.1111/ejn.16349, PMID: 38646841

[ref40] Luhmann H. J. Fukuda A. Kilb W. (2015). Control of cortical neuronal migration by glutamate and GABA. Front. Cell. Neurosci. 9:4. doi: 10.3389/fncel.2015.00004, PMID: 25688185 PMC4311642

[ref41] Minardi R. Licchetta L. Baroni M. C. Pippucci T. Stipa C. Mostacci B.. (2020). Whole-exome sequencing in adult patients with developmental and epileptic encephalopathy: it is never too late. Clin. Genet. 98, 477–485. doi: 10.1111/cge.13823, PMID: 32725632

[ref42] Mizuno E. Kitamura N. Komada M. (2007). 14-3-3-dependent inhibition of the deubiquitinating activity of UBPY and its cancellation in the M phase. Exp. Cell Res. 313, 3624–3634. doi: 10.1016/j.yexcr.2007.07.028, PMID: 17720156

[ref43] Moore B. W. (1969). “Acidic Proteins” in Chemical architecture of the nervous system. ed. Lajtha A. (Boston, MA: Springer US), 93–99.

[ref44] Moore B. W. McGregor D. (1965). Chromatographic and electrophoretic fractionation of soluble proteins of brain and liver. J. Biol. Chem. 240, 1647–1653. doi: 10.1016/S0021-9258(18)97483-1, PMID: 14285503

[ref45] Mukhopadhyay A. Sehgal L. Bose A. Gulvady A. Senapati P. Thorat R.. (2016). 14-3-3γ prevents centrosome amplification and neoplastic progression. Sci. Rep. 6:26580. doi: 10.1038/srep26580, PMID: 27253419 PMC4890593

[ref46] Obsil T. Obsilova V. (2011). Structural basis of 14-3-3 protein functions. Semin. Cell Dev. Biol. 22, 663–672. doi: 10.1016/j.semcdb.2011.09.001, PMID: 21920446

[ref47] Obsilova V. Obsil T. (2022). Structural insights into the functional roles of 14-3-3 proteins. Front. Mol. Biosci. 9:1016071. doi: 10.3389/fmolb.2022.1016071, PMID: 36188227 PMC9523730

[ref48] Oh S.-J. Woo J. Lee Y.-S. Cho M. Kim E. Cho N.-C.. (2017). Direct interaction with 14-3-3γ promotes surface expression of best 1 channel in astrocyte. Mol. Brain 10:51. doi: 10.1186/s13041-017-0331-x, PMID: 29121962 PMC5679146

[ref49] Pagliuso A. Valente C. Giordano L. L. Filograna A. Li G. Circolo D.. (2016). Golgi membrane fission requires the CtBP1-S/BARS-induced activation of lysophosphatidic acid acyltransferase δ. Nat. Commun. 7:12148. doi: 10.1038/ncomms12148, PMID: 27401954 PMC4945875

[ref50] Peyrl A. Weitzdoerfer R. Gulesserian T. Fountoulakis M. Lubec G. (2002). Aberrant expression of signaling-related proteins 14-3-3 gamma and RACK1 in fetal down syndrome brain (trisomy 21). Electrophoresis 23, 152–157. doi: 10.1002/1522-2683(200201)23:1<152::AID-ELPS152>3.0.CO;2-T, PMID: 11824616

[ref51] Qian Chen X. Cheung Hoi Yu A. (2002). The association of 14-3-3γ and actin plays a role in cell division and apoptosis in astrocytes. Biochem. Biophys. Res. Commun. 296, 657–663. doi: 10.1016/S0006-291X(02)00895-1, PMID: 12176032

[ref52] Qiao H. Foote M. Graham K. Wu Y. Zhou Y. (2014). 14-3-3 proteins are required for hippocampal long-term potentiation and associative learning and memory. J. Neurosci. 34, 4801–4808. doi: 10.1523/JNEUROSCI.4393-13.2014, PMID: 24695700 PMC3972712

[ref53] Roy D. S. Zhang Y. Aida T. Choi S. Chen Q. Hou Y.. (2021). Anterior thalamic dysfunction underlies cognitive deficits in a subset of neuropsychiatric disease models. Neuron 109, 2590–2603. doi: 10.1016/j.neuron.2021.06.005, PMID: 34197733 PMC8376805

[ref54] Sarlo G. L. Holton K. F. (2021). Brain concentrations of glutamate and GABA in human epilepsy: a review. Seizure 91, 213–227. doi: 10.1016/j.seizure.2021.06.028, PMID: 34233236

[ref55] Schindler C. K. Heverin M. Henshall D. C. (2006). Isoform- and subcellular fraction-specific differences in hippocampal 14-3-3 levels following experimentally evoked seizures and in human temporal lobe epilepsy. J. Neurochem. 99, 561–569. doi: 10.1111/j.1471-4159.2006.04153.x, PMID: 16981892

[ref56] Sedláčková L. Štěrbová K. Vlčková M. Maulisová A. Laššuthová P. (2021). A novel variant in YWHAG further supports phenotype of developmental and epileptic encephalopathy. Am. J. Med. Genet. A 185, 1363–1365. doi: 10.1002/ajmg.a.62116, PMID: 33590706

[ref57] Sehgal L. Mukhopadhyay A. Rajan A. Khapare N. Sawant M. Vishal S. S.. (2014). 14-3-3γ meditated transport of plakoglobin to the cell border is required for the initiation of desmosome assembly in vitro and in vivo. J. Cell Sci. 127, 2174–2188. doi: 10.1242/jcs.125807, PMID: 24610948

[ref58] Sengupta A. Liriano J. Bienkiewicz E. A. Miller B. G. Frederich J. H. (2020). Probing the 14-3-3 isoform-specificity profile of protein–protein interactions stabilized by Fusicoccin a. ACS Omega 5, 25029–25035. doi: 10.1021/acsomega.0c01454, PMID: 33043180 PMC7542595

[ref59] Shen Y. H. Godlewski J. Bronisz A. Zhu J. Comb M. J. Avruch J.. (2003). Significance of 14-3-3 self-dimerization for phosphorylation-dependent target binding. Mol. Biol. Cell 14, 4721–4733. doi: 10.1091/mbc.e02-12-0821, PMID: 14551260 PMC266786

[ref60] Skjevik Å. A. Mileni M. Baumann A. Halskau Ø. Teigen K. Stevens R. C.. (2014). The N-terminal sequence of tyrosine hydroxylase is a Conformationally versatile motif that binds 14-3-3 proteins and membranes. J. Mol. Biol. 426, 150–168. doi: 10.1016/j.jmb.2013.09.012, PMID: 24055376 PMC3872242

[ref61] Stern T. Orenstein N. Fellner A. Lev-El Halabi N. Shuldiner A. R. Gonzaga-Jauregui C.. (2021). Epilepsy and electroencephalogram evolution in *YWHAG* gene mutation: a new phenotype and review of the literature. Am. J. Med. Genet. A 185, 901–908. doi: 10.1002/ajmg.a.62026, PMID: 33393734

[ref62] Stevers L. M. Sijbesma E. Botta M. MacKintosh C. Obsil T. Landrieu I.. (2018). Modulators of 14-3-3 protein–protein interactions. J. Med. Chem. 61, 3755–3778. doi: 10.1021/acs.jmedchem.7b00574, PMID: 28968506 PMC5949722

[ref63] Tan Q. Cheng M. Yang Y. Wang T. Ouyang S. Liu C.. (2025). The phenotypic spectrum of *YWHAG*-related epilepsy: from mild febrile seizures to severe developmental delay and epileptic encephalopathy. Dev. Med. Child Neurol. doi: 10.1111/dmcn.16320, PMID: 40186408

[ref64] Tian T. Cao X. Chen Y. Jin L. Li Z. Han X.. (2021). Somatic and *de novo* germline variants of MEDs in human neural tube defects. Front. Cell Dev. Biol. 9:641831. doi: 10.3389/fcell.2021.641831, PMID: 33748132 PMC7969791

[ref65] Valente C. Turacchio G. Mariggiò S. Pagliuso A. Gaibisso R. Di Tullio G.. (2012). A 14-3-3γ dimer-based scaffold bridges CtBP1-S/BARS to PI (4) KIIIβ to regulate post-Golgi carrier formation. Nat. Cell Biol. 14, 343–354. doi: 10.1038/ncb2445, PMID: 22366688

[ref66] Visser E. J. Jaishankar P. Sijbesma E. Pennings M. A. M. Vandenboorn E. M. F. Guillory X.. (2023). From tethered to freestanding stabilizers of 14-3-3 protein-protein interactions through fragment linking. Angew. Chem. Int. Ed. 62:e202308004. doi: 10.1002/anie.202308004, PMID: 37455289 PMC11287480

[ref67] Wachi T. Cornell B. Marshall C. Zhukarev V. Baas P. W. Toyo-oka K. (2016). Ablation of the 14-3-3gamma protein results in neuronal migration delay and morphological defects in the developing cerebral cortex. Dev. Neurobiol. 76, 600–614. doi: 10.1002/dneu.22335, PMID: 26297819

[ref68] Watanabe M. Isobe T. Ichimura T. Kuwano R. Takahashi Y. Kondo H. (1993). Molecular cloning of rat cDNAs for β and γ subtypes of 14-3-3 protein and developmental changes in expression of their mRNAs in the nervous system. Mol. Brain Res. 17, 135–146. doi: 10.1016/0169-328X(93)90082-Z, PMID: 8381897

[ref69] Wen Y. Zhang G. Liu L. Zhang P. Lin L. Mei R.. (2022). HAP1 interacts with 14-3-3 to regulate epileptic seizure via GABAAR-mediated inhibitory synaptic transmission in pentylenetetrazole rat model. Neurosci. Res. 182, 7–14. doi: 10.1016/j.neures.2022.05.006, PMID: 35609730

[ref70] Wiltfang J. Otto M. Baxter H. C. Bodemer M. Steinacker P. Bahn E.. (1999). Isoform pattern of 14-3-3 proteins in the cerebrospinal fluid of patients with Creutzfeldt-Jakob disease. J. Neurochem. 73, 2485–2490. doi: 10.1046/j.1471-4159.1999.0732485.x, PMID: 10582609

[ref71] Xu J. Wang J. He Z. Chen P. Jiang X. Chen Y.. (2021). LncRNA CERS6-AS1 promotes proliferation and metastasis through the upregulation of YWHAG and activation of ERK signaling in pancreatic cancer. Cell Death Dis. 12:648. doi: 10.1038/s41419-021-03921-3, PMID: 34168120 PMC8225895

[ref72] Yang X. Lee W. H. Sobott F. Papagrigoriou E. Robinson C. V. Grossmann J. G.. (2006). Structural basis for protein–protein interactions in the 14-3-3 protein family. Proc. Natl. Acad. Sci. 103, 17237–17242. doi: 10.1073/pnas.0605779103, PMID: 17085597 PMC1859916

[ref73] Ye X.-G. Liu Z.-G. Wang J. Dai J.-M. Qiao P.-X. Gao P.-M.. (2021). YWHAG mutations cause childhood myoclonic epilepsy and febrile seizures: molecular sub-regional effect and mechanism. Front. Genet. 12:632466. doi: 10.3389/fgene.2021.632466, PMID: 33767733 PMC7985244

[ref74] Yi Z. Song Z. Xue J. Yang C. Li F. Pan H.. (2022). A heterozygous missense variant in the YWHAG gene causing developmental and epileptic encephalopathy 56 in a Chinese family. BMC Med. Genet. 15:216. doi: 10.1186/s12920-022-01377-8, PMID: 36243722 PMC9569127

[ref75] Zhang J. Navarrete M. Wu Y. Zhou Y. (2022). 14-3-3 dysfunction in dorsal hippocampus CA1 (dCA1) induces psychomotor behavior via a dCA1-lateral septum-ventral tegmental area pathway. Front. Mol. Neurosci. 15:817227. doi: 10.3389/fnmol.2022.817227, PMID: 35237127 PMC8882652

