# Correction: Role of the YWHAG gene mutations in developmental and epileptic encephalopathy

**DOI:** 10.3389/fnins.2025.1694725

**Published:** 2025-09-22

**Authors:** Violet Vilmont, Richard S. Nowakowski, Yi Zhou

**Affiliations:** Department of Biomedical Sciences, Florida State University College of Medicine, Tallahassee, FL, United States

**Keywords:** YWHAG mutation, 14-3-3γ protein, developmental and epileptic encephalopathy, epilepsy, seizure, neuronal hyperexcitability, DEE, 14-3-3 protein family

There was a mistake in the caption of Figure 6 as published. There was a mistake at the end of the Figure 6 legend, stating the source of the Figure 6. The statement “Source: Logue et al. (2024)” is incorrect, and must be replaced with “Created in Biorender”. The corrected caption of Figure 6 appears below.

There was a mistake in the caption of Figure 7 as published. There was a mistake at the end of the Figure 7 legend, stating how Figure 7 was created. The statement “Created in Biorender” is incorrect, and must be removed. Figure 7 was not created in Biorender, it was a reference figure taken from Logue et al. (2024). The corrected caption of Figure 7 appears below.

The figures 1 and 2 were in the wrong order, and the figures 6 and 7 were in the wrong order. The images of Figure 1 and Figure 2 are incorrect and must be switched with each other; the figure captions and legends are correct as published. The images of Figures 6 and 7 are incorrect and must be switched with each other. The figure order has now been corrected.

**Figure 1:**
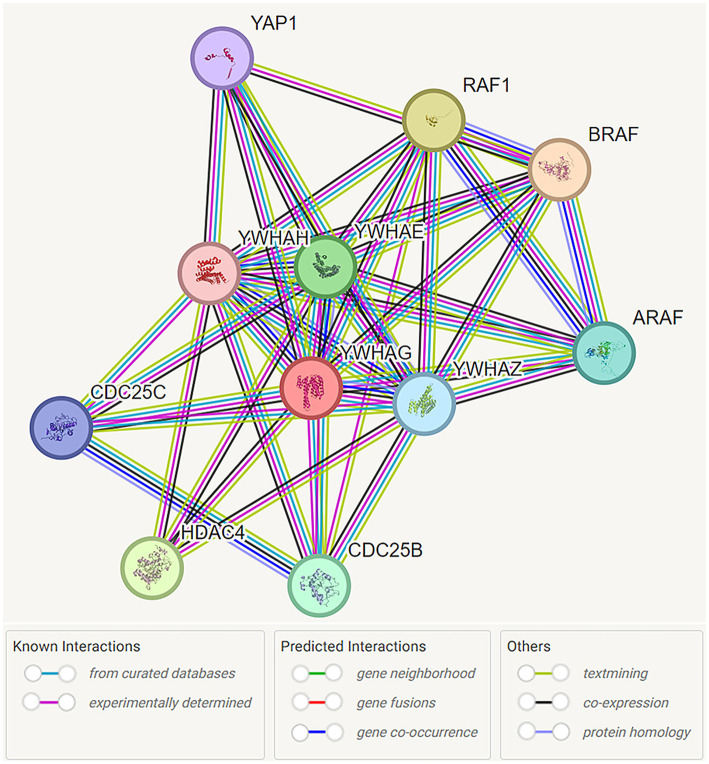
Functional interaction network of 14-3-3γ encoded by YWHAG. Network nodes are labeled with the name of the individual genes which encode the represented proteins. Protein interactions are represented by color coded lines, based on known and predicted interactions, as indicated by the legend. Source: https://stringdb.org/cgi/network?taskId=bMYGzw1kOtuv&sessionId=bMliKFIeKj5g. Screenshot image obtained from the STRING database (string-db.org). Licensed under the Creative Commons Attribution 4.0 International License (CC BY 4.0).

**Figure 2:**
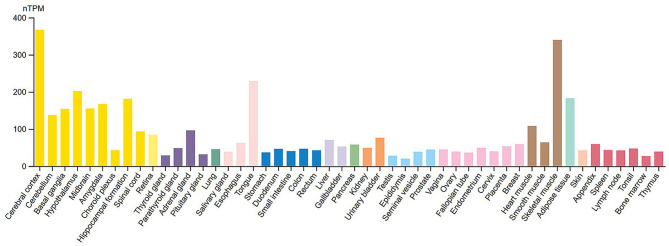
RNA tissue specificity expression of 14-3-3γ. Normalized RNA expression levels (nTPM) shown for 55 tissue types. Color coding is based on tissue groups, each consisting of tissues with functional features in common. RNA tissue specificity expression is enhanced in brain (yellow bars) and skeletal muscle cells (brown bars). Source: https://www.proteinatlas.org/ENSG00000170027-YWHAG/tissue. Screenshot image obtained from the Human Protein Atlas (proteinatlas.org). Licensed under the Creative Commons Attribution-ShareAlike 4.0 International License (CC BY-SA 4.0).

**Figure 6:**
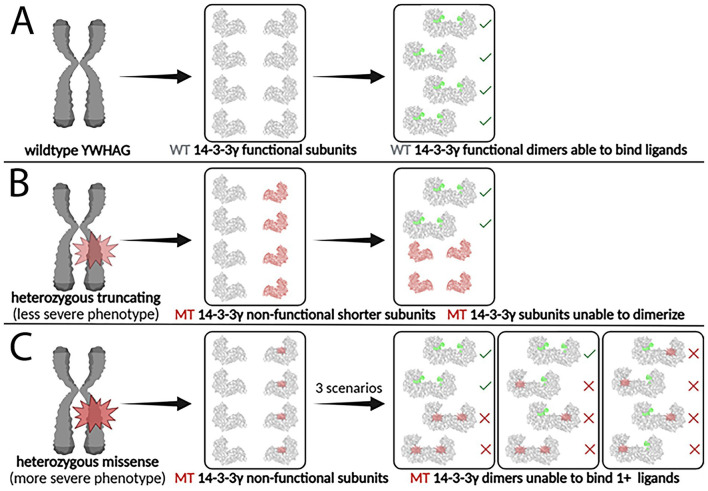
Impact of heterozygous truncating and missense YWHAG mutations on 14-3-3γ dimer formation and function. **(A)** The wildtype YWHAG gene produces normal 14-3-3γ subunits, which assemble into functional 14-3-3γ dimers, able to bind phosphorylated ligands. **(B)** A truncating mutation produces non-functional mutant 14-3-3γ subunits that are shorter and smaller, which are unable to dimerize and to bind phosphorylated ligands. **(C)** A missense mutation produces non-functional mutant 14-3-3γ subunits that are the same size as the wildtype subunits, which are able to dimerize but unable to bind two phosphorylated ligands. WT, wildtype; MT, mutant. Created in Biorender.

**Figure 7:**
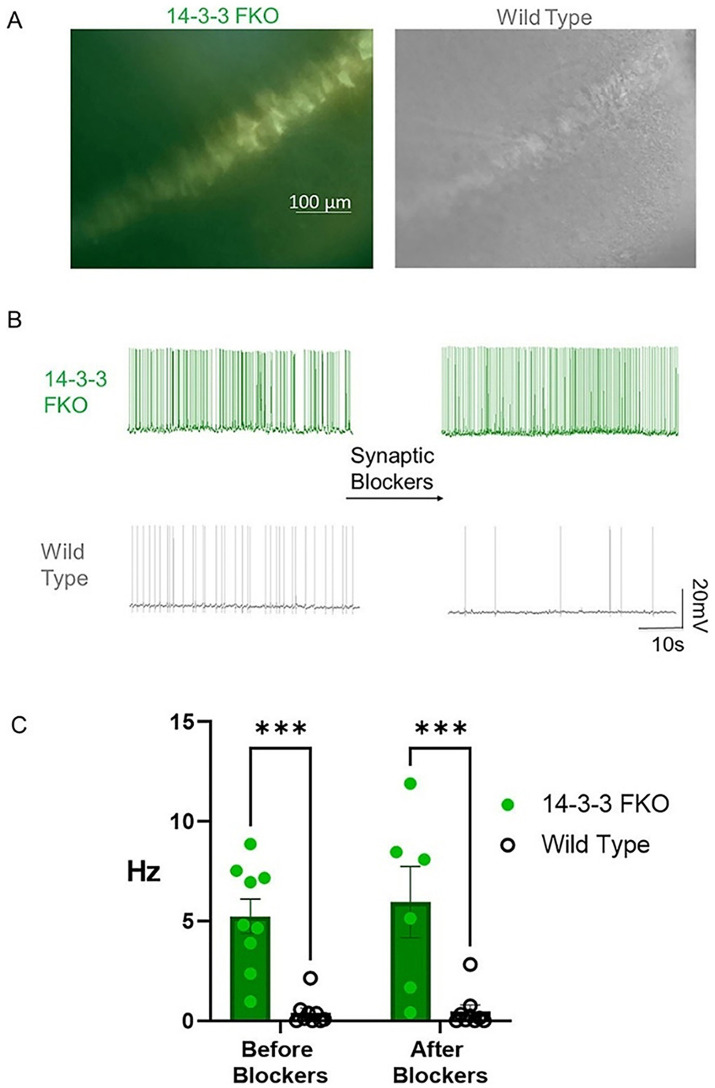
14-3-3 FKO hippocampal CA1 neurons fire more APs than WT neurons in the presence and absence of synaptic blockers. **(A)** Hippocampal slice images captured using phase contrast and fluorescence microscopy show 14-3-3 FKO neurons (left) identified by their YFP fluorescence, indicating difopein expression. **(B)** Traces of spontaneous AP firing in 14-3-3 FKO and WT neurons under whole-cell configuration, before and after synaptic blocker application. **(C)** Group data showing a higher AP firing rate for 14-3-3 FKO cells (*n* = 9 before blockers, 6 after blockers) than WT cells (*n* = 9 before blockers, 8 after blockers). AP, action potential; FKO, functional knockout; WT, wildtype; CA1, one of four hippocampal subfields that make up hippocampus structure. Source: Figure 1 from Logue et al. (2024).

The original version of this article has been updated.

